# Effects of a brief resonance frequency breathing intervention on heart rate variability and perceived stress in postgraduate students: a pilot and feasibility study

**DOI:** 10.3389/fpsyg.2026.1787922

**Published:** 2026-10-07

**Authors:** Alessandro Spizzirri, Neil Buchanan

**Affiliations:** Moray House School of Education and Sport, University of Edinburgh, Edinburgh, United Kingdom

**Keywords:** resonance frequency breathing, heart rate variability, perceived stress, postgraduate students, HRV biofeedback, autonomic regulation, pilot and feasibility study

## Abstract

**Introduction:**

Stress during postgraduate education can undermine well-being, academic performance, and physiological regulation. Resonance frequency breathing (RFB) is a slow-paced breathing technique associated with enhanced autonomic regulation, as indexed by heart rate variability (HRV). However, validated protocols are often time-intensive, limiting real-world implementation. This pilot study evaluated the preliminary effects, feasibility, and acceptability of a brief, app-guided RFB intervention during an academically demanding period.

**Methods:**

Twelve postgraduate students were randomly assigned to an RFB intervention group (*n* = 6) or a no-intervention control group (*n* = 6). Intervention participants completed daily 10-minute sessions for three weeks at their individually determined resonance frequency. The standard deviation of normal-to-normal intervals (SDNN), root mean square of successive differences (RMSSD), and perceived stress, measured using a PSS-10 adapted to a one-week recall period, were assessed weekly. Post-intervention qualitative interviews with five intervention participants explored feasibility, perceived impact, engagement, and future application.

**Results:**

Between-group comparisons of change scores showed significant differences for SDNN at Weeks 2 (*p* = 0.004, *r* = 0.94) and 3 (*p* = 0.026, *r* = 0.78), and for RMSSD at Week 3 (*p* = 0.026, *r* = 0.78) with values increasing in the RFB group and declining in controls. No significant between-group difference in perceived stress was observed. By Week 3, however, the intervention group showed a mean decrease of 6.00 points from baseline, compared with a 4.50-point increase among controls (*r* = −0.47, *p* = 0.199). Interviewed participants generally experienced the protocol as manageable, reported perceived benefits for stress coping, and expressed interest in continuing RFB.

**Discussion:**

In this pilot sample, the brief RFB protocol was feasible and acceptable and was associated with increases in resting HRV relative to controls during heightened academic demand. The study did not demonstrate a significant between-group difference in perceived stress. Given the small sample, short duration, and absence of an active control, the findings are preliminary. Larger controlled trials with follow-up assessments are needed to evaluate efficacy, durability, and real-world implementation.

## 1 Introduction

Postgraduate study requires sustained engagement in research, coursework, and, for many, teaching responsibilities (Offstein et al., 2004). Accumulating evidence indicates that postgraduate students experience high levels of psychological distress. A large international survey reported that approximately 41% of respondents met criteria for moderate-to-severe anxiety symptoms and 39% for depression (Evans et al., 2018). While direct comparison with general population rates has been debated (Duffy et al., 2019), these findings are broadly consistent with evidence of elevated psychological distress in postgraduate student populations. Stress among postgraduate students has also been associated with the core dimensions of burnout, including exhaustion, cynicism, and reduced academic efficacy (Allen et al., 2021). Despite recognition of these concerns, institutional support services remain limited. Short-term counselling interventions can be effective (Biasi et al., 2017), but rising demand, limited service capacity, and variation in provision can constrain their reach (Broglia et al., 2018). Engagement with available services may be further limited by waiting times (Broglia et al., 2018) and perceived stigma (Osborn et al., 2022). These limitations underscore the need for targeted, scalable, and low-burden strategies that can support stress regulation during phases of heightened demand.

### 1.1 Foundations of the stress response

Effective intervention development requires an understanding of the mechanisms underlying the stress response, as these represent potential targets for regulation. Stress is a psychophysiological process involving interacting autonomic and neuroendocrine systems. The autonomic nervous system (ANS) comprises sympathetic (SNS) and parasympathetic (PNS) branches (Ghasemi et al., 2024). Acute stress activates the SNS, facilitating mobilisation through “fight-or-flight” responses, whereas the PNS supports recovery through “rest-and-digest” processes. Adaptive stress regulation depends on flexible coordination between these systems. However, prolonged stress exposure can disrupt this balance, resulting in sustained sympathetic activation, reduced parasympathetic influence, and dysregulation of the hypothalamic–pituitary–adrenal (HPA) axis (Ghasemi et al., 2024). Polyvagal Theory conceptualises the ANS as a hierarchically organised system comprising ventral vagal, sympathetic, and dorsal vagal pathways that support dynamic transitions between states of safety, mobilisation, and withdrawal (Porges, 2007). Vagal afferent and efferent pathways support bidirectional communication between peripheral organs and central neural structures, enabling rapid physiological adaptation to environmental demands. Complementing this framework, the Neurovisceral Integration Model emphasises the role of prefrontal–limbic networks in regulating vagal tone and coordinating cognitive, emotional, and physiological processes (Thayer and Lane, 2000). Chronic stress can alter the structure and function of neural circuits that regulate HPA-axis and autonomic stress responses, thereby affecting adaptive responding (Ulrich-Lai and Herman, 2009).

### 1.2 Heart rate variability as an index of autonomic regulation

Heart rate variability (HRV)—the variation in time between successive heartbeats—serves as a non-invasive index of autonomic flexibility and is influenced by vagal function, reflecting the capacity of the cardiovascular system to dynamically adapt to internal and external demands (Shaffer and Ginsberg, 2017). Higher HRV is typically associated with efficient parasympathetic control, greater physiological adaptability, and enhanced executive functioning, emotion regulation, and stress resilience (Mather and Thayer, 2018; Magnon et al., 2022). In contrast, lower HRV has been linked to stress-related psychopathology, including anxiety disorders, depression, and PTSD (Chalmers et al., 2014; Beauchaine and Thayer, 2015; Huang et al., 2018), as well as poorer performance on tasks involving executive control and response inhibition (Thayer et al., 2009; Hansen et al., 2004; Krypotos et al., 2011). Individuals reporting higher levels of chronic stress tended to show lower resting HRV (Jiryis et al., 2022), a pattern suggesting less flexible autonomic regulation (Shaffer and Ginsberg, 2017). In student populations, HRV has been shown to decrease during examination periods and recover once stress subsides (Hammoud et al., 2019). Similarly, Kazdagli et al. (2024) found that students with higher perceived exam anxiety showed elevated electrophysiological stress indicators, including stress-related HRV indices and electrodermal activity, on exam days; these measures were negatively correlated with exam scores. Benítez-Agudelo et al. (2025) observed non-significant downward trends in several HRV indices across an academic semester alongside significant increases in anxiety and depressive symptoms, while higher baseline HRV predicted better academic performance. Together, these findings suggest that autonomic regulation may be both undermined by and functionally relevant to university demands. Despite this evidence, limited research has directly evaluated HRV as a modifiable target in postgraduate samples or examined whether autonomic recovery can be supported during intensive academic phases.

### 1.3 Baroreflex and respiratory sinus arrhythmia

HRV reflects the interaction of multiple autonomic mechanisms, most notably respiratory sinus arrhythmia (RSA) and baroreflex sensitivity. RSA refers to the rhythmic acceleration of heart rate during inhalation and deceleration during exhalation, a process mediated primarily by parasympathetic vagal activity, reflecting coordinated respiratory–cardiovascular coupling (Shaffer and Ginsberg, 2017). The baroreflex functions as a negative feedback system that stabilises arterial blood pressure by dynamically adjusting heart rate in response to pressure fluctuations (Lehrer et al., 2003). Respiratory-driven changes in intrathoracic pressure produce oscillations in arterial pressure. During inhalation, cardiac vagal outflow decreases and heart rate rises, whereas during exhalation, vagal outflow is restored and heart rate slows. Arterial baroreceptors detect the associated pressure fluctuations and relay afferent signals via the glossopharyngeal and vagus nerves to the nucleus tractus solitarius in the medulla, which coordinates autonomic adjustments in heart rate and vascular tone (Shaffer and Meehan, 2020). The coordinated interaction between RSA and the baroreflex enables rapid, moment-to-moment adjustment of autonomic output and contributes substantially to short-term HRV. Importantly, because both mechanisms are shaped by respiratory dynamics, breathing represents a direct and accessible means of modulating RSA amplitude and baroreflex function.

### 1.4 Breathing-based approaches to autonomic regulation

Breathing-based approaches have been widely investigated as practical methods for influencing autonomic regulation and reducing stress. These interventions are non-invasive, cost-effective, and readily learned, contributing to their application across clinical and non-clinical contexts. Broadly, breathing-based interventions can be categorised into breath-awareness and breath-control practices (Vlemincx and Cortez-Vázquez, 2024). Breath-awareness approaches, such as mindful breathing and breath-focused meditation, primarily target attentional regulation and reductions in cognitive rumination. In contrast, breath-control techniques involve deliberate manipulation of respiratory parameters, including rate, depth, rhythm, or inhalation–exhalation ratios. Slow breathing—typically defined as fewer than 10 breaths per minute—is the most extensively studied form of breath control and has been associated with improvements in HRV, blood pressure, and psychological outcomes, including anxiety and perceived stress (Lehrer and Gevirtz, 2014; Lin et al., 2023; Goessl et al., 2017). However, effects vary across studies, and differences in breathing parameters can elicit divergent autonomic responses (Lehrer and Gevirtz, 2014; Song and Lehrer, 2003). These findings have prompted increasing interest in more precise, mechanism-driven breathing protocols.

### 1.5 Resonance frequency breathing: mechanisms and applications

Resonance frequency breathing (RFB) is a slow-paced breathing technique that trains individuals to breathe at approximately 0.1 Hz—typically between 4.5 and 7.0 breaths per minute—to optimise synchrony between RSA and baroreflex oscillations (Lehrer et al., 2003; Vaschillo et al., 2002). At this frequency, respiratory and cardiovascular rhythms align to produce a resonance effect, resulting in maximal HRV amplitude (Vaschillo et al., 2002). Repeated RFB practice has been shown to enhance baroreflex sensitivity, and support adaptive recovery from stress (Lehrer et al., 2003; Lehrer and Gevirtz, 2014). Meta-analytic evidence indicates that HRV biofeedback is associated with large reductions in self-reported stress and anxiety, both from pre- to post-intervention (Hedges’ g = 0.81) and relative to control conditions (Hedges’ g = 0.83; Goessl et al., 2017). Beyond laboratory settings, RFB has been examined in academic examination and musical-performance contexts, with reductions in physiological arousal reported in these settings (Deschodt-Arsac et al., 2018; Wells et al., 2012). RFB can be delivered through several formats (Lalanza et al., 2023). Personalised optimisation involves identifying each participant’s resonance frequency (RF), defined as the breathing rate that maximises respiratory-linked heart-rate oscillations, by comparing responses across several paced-breathing trials (Shaffer and Meehan, 2020). Adaptive biofeedback uses real-time cardiovascular data to guide participants toward their RF, whereas preset pacing applies a fixed breathing rate, usually six breaths per minute, which may not correspond to each participant’s RF (Lalanza et al., 2023; Shaffer and Meehan, 2020).

### 1.6 Challenges in scalability and adherence

Despite its promise, broader implementation of RFB faces practical challenges. Many established protocols recommend 20-min sessions performed twice daily to achieve sustained effects (Lehrer et al., 2000, 2013). Findings from less intensive interventions remain mixed. For example, Chaitanya et al. (2022) reported improvements in HRV and perceived stress following a four-week intervention involving a single daily 20-min session, whereas Groß and Kohlmann (2021) observed no HRV changes following a five-minute daily protocol. These inconsistencies highlight unresolved questions regarding minimal effective dosing. Adherence represents an additional challenge, particularly in remote and self-guided formats. Competing occupational and personal demands interfere with consistent practice (Vlemincx and Cortez-Vázquez, 2024), and attrition rates remain high even in accessible digital interventions. In a feasibility study of remote, self-administered HRV biofeedback among healthcare workers, Hirten et al. (2024) found that only 16.5% of participants achieved at least 50% compliance. These findings suggest that effectiveness depends not only on physiological mechanisms but also on perceived value, usability, and integration into daily routines.

### 1.7 Current gaps and rationale for the present study

The present study evaluated the feasibility and preliminary effects of a brief, remotely delivered RFB intervention designed to support physiological and psychological stress regulation in postgraduate students. Longitudinal research suggests that the academic semester may be accompanied by worsening mental health symptoms and changes in autonomic regulation. Benítez-Agudelo et al. (2025) observed significant increases in anxiety and depressive symptoms across a semester; several HRV indices showed small downward changes, although these were not statistically significant after correction. Higher baseline HRV predicted better academic performance, highlighting autonomic regulation as a potentially relevant target for supporting student well-being and academic functioning. Although RFB has demonstrated effects on HRV, many validated protocols are time-intensive, limiting their application in academically demanding contexts. To address this gap, the current study adapted the protocol described by Chaitanya et al. (2022) by implementing a 10-min daily intervention over 3 weeks, rather than a 20-min, four-week format. The intervention incorporated individualised RF determination, consistent with established protocols designed to identify the breathing rate that maximises each participant’s HRV response (Shaffer and Meehan, 2020). Qualitative feedback was integrated to assess protocol feasibility (Creswell and Creswell, 2018). Importantly, the intervention was delivered during the postgraduate dissertation semester, providing an ecologically meaningful context in which to evaluate stress-related outcomes.

### 1.8 Objectives and hypotheses

This study pursued four objectives. First, it examined the physiological effects of a three-week RFB intervention by assessing changes in HRV indices. Second, it evaluated psychological outcomes by examining changes in perceived stress. Third, it explored associations between physiological and psychological changes by assessing correlations between HRV and perceived stress. Finally, it investigated feasibility, engagement, perceived impact, and potential future application through qualitative interviews. It was hypothesised that: (H1) participants in the RFB group would demonstrate greater increases in HRV (SDNN and RMSSD) relative to controls; (H2) participants in the RFB group would report greater reductions in perceived stress; (H3) increases in HRV would be negatively associated with changes in perceived stress; and (H4) participants would report the intervention as feasible, acceptable, and beneficial.

## 2 Methods

### 2.1 Study design

A two-arm, randomised, mixed-methods pilot and feasibility trial was conducted to examine the effects of a brief RFB intervention on physiological and psychological outcomes. The study was designed to prioritise the evaluation of procedural feasibility, intervention acceptability, and data collection processes rather than to provide definitive hypothesis testing (Lancaster et al., 2004; Thabane et al., 2010). Accordingly, no formal *a priori* power calculation was conducted; pilot samples are intentionally small and serve to generate preliminary parameter estimates and inform the design of a subsequent adequately powered trial (Eldridge et al., 2016; Whitehead et al., 2016). Importantly, HRV biofeedback interventions have been associated with large effect sizes in prior research (Goessl et al., 2017; Lehrer and Gevirtz, 2014), suggesting that preliminary effects were likely to be detectable within this small sample, although more nuanced effects may have remained undetected. Following Creswell and Creswell’s (2018) mixed-methods framework, the study adopted a convergent parallel design, whereby quantitative and qualitative data were collected during the same phase, analysed independently, and integrated during interpretation to allow findings from both strands to inform one another. Due to the nature of the behavioural intervention, participants were aware of their group allocation; however, primary physiological outcomes (HRV) were recorded automatically by the monitoring devices, thereby reducing the risk of assessor bias in the primary outcome.

### 2.2 Participants

Twelve postgraduate students (8 females, 4 males; *M* = 24.0, SD = 1.81 years, range = 22–28) were recruited from the University of Edinburgh using convenience sampling (Etikan et al., 2016). Participants were assigned to either a resonance frequency breathing (RFB) intervention group (*n* = 6) or a control group (*n* = 6) using a computer-generated randomisation sequence (Microsoft Excel). Baseline comparability between groups was assessed via a Mann–Whitney U test and Fisher’s exact test, which indicated no significant differences in age (*p* = 0.684) or gender (*p* = 1.000). Participant demographic characteristics are presented in Table 1.

**Table 1:** Participant characteristics.

Variable	Intervention (*n* = 6)	Control (*n* = 6)	Total (*n* = 12)	*p*
Age, M (SD), range	24.3 (2.07), 22–28	23.7 (1.63), 22–26	24.0 (1.81), 22–28	0.684
Gender, n (%)				1.000
Female	4 (66.7)	4 (66.7)	8 (66.7)	
Male	2 (33.3)	2 (33.3)	4 (33.3)	

Data are presented as M (SD) unless otherwise indicated. *p* values are from a Mann-Whitney *U* test (age) and Fisher’s exact test (gender). No significant between-group differences were observed at baseline.

Inclusion criteria were: (i) current postgraduate enrolment, (ii) age 18–35 years, and (iii) ownership of a smartphone capable of running the breathing application. Exclusion criteria (informed by Kim et al., 2018) included: (i) regular engagement in meditation, yoga, or breathwork; (ii) cardiovascular, neurological, or psychiatric disorders or medications influencing autonomic function; (iii) smoking, alcohol consumption > 14 units per week, or recreational drug use; and (iv) body-mass index < 18 or > 25 kg/m^2^.

Baseline comparisons using Mann–Whitney *U* tests revealed no significant between-group differences on any primary outcome measure prior to commencing the intervention (see Table 2). Nonsignificant baseline tests do not establish equivalence.

**Table 2:** Baseline group comparisons across primary outcome measures.

Measure	Intervention, M (SD)	Control, M (SD)	*U*	*p*
SDNN (ms)	74.15 (33.95)	89.90 (41.61)	13.00	0.485
RMSSD (ms)	43.51 (30.01)	64.95 (46.19)	15.00	0.699
PSS-10	15.50 (11.57)	12.50 (2.95)	16.50	0.872

Data are presented as M (SD). No significant between-group differences were observed at baseline for any primary outcome measure (all *p* > 0.05).

### 2.3 Instruments and measures

#### 2.3.1 Physiological assessment

HRV was recorded using the Polar H10 chest-strap monitor (Polar Electro Oy, Kempele, Finland) used in conjunction with the Elite HRV smartphone application (Elite HRV, Asheville, NC, United States). The Polar H10 captured R–R intervals at a sampling rate of 1,000 Hz, while Elite HRV provided signal visualisation and artefact detection. Recordings were obtained over 5 min under seated resting conditions, in line with Task Force of the European Society of Cardiology and the North American Society of Pacing and Electrophysiology (1996) recommendations for short-term HRV analysis. Only recordings with an artefact-correction score of 0 (indicating signal stability and absence of ectopic beats or motion artefacts) were retained for analysis. Two time-domain HRV indices were extracted: SDNN (standard deviation of normal-to-normal intervals), reflecting overall autonomic variability, and RMSSD (root mean square of successive differences), indexing short-term parasympathetic modulation (Laborde et al., 2017). In short-term recordings (e.g., 5 min), SDNN reflects variability arising from both sympathetic and parasympathetic inputs and is substantially influenced by vagal activity at this timescale, whereas RMSSD more directly reflects vagally mediated cardiac control and is relatively less influenced by sympathetic activity under resting conditions. Together, these indices capture complementary aspects of autonomic regulation relevant to the baroreflex-mediated mechanisms underlying RFB.

#### 2.3.2 Psychological assessment

Perceived stress was assessed using the 10-item version of the Perceived Stress Scale (PSS-10; Cohen and Williamson, 1988), derived from the original 14-item PSS (Cohen et al., 1983). To align with the three-week intervention and weekly assessment schedule, the original one-month reference frame was adapted to 1 week. This modification is consistent with previous research implementing shorter PSS recall intervals (Murray et al., 2023), and is supported by the scale’s developer, who notes that shorter retrospective periods are unlikely to compromise measurement integrity (Cohen, 2026), though its potential influence on measurement sensitivity is acknowledged as a limitation. Items were rated on a 5-point Likert scale (0 = never to 4 = very often), producing total scores from 0 to 40, with higher scores indicating greater perceived stress. The PSS-10 demonstrates robust internal reliability across diverse populations, with Cronbach’s alpha exceeding 0.70 across studies (Lee, 2012).

### 2.4 Procedure

The schedule of research activities is summarised in Table 3.

**Table 3:** Schedule of research activities.

Phase	Week	Activity
Familiarisation	Week 0	Baseline HRV; Baseline PSS-10 (both groups); RF assessment (intervention group)
Training	Weeks 1–3	Daily RF Breathing (intervention group); HRV Recording Mon/Wed/Fri and PSS-10 Fri (both groups)
Follow-up	Week 4	Semi-structured interview (intervention group)

#### 2.4.1 Phase 1: Familiarisation (week 0)

All participants attended an individual familiarisation session of approximately 45 min prior to the intervention period. During this session, participants received an overview of the study protocol, including aims, timelines, and data submission procedures, and provided written informed consent. Participants were trained to use the Polar H10 chest-strap monitor in conjunction with the Elite HRV application to obtain seated resting-state HRV recordings, and baseline measurements were obtained under standardised conditions. Instructions were also provided for submitting HRV recordings via Microsoft Teams and completing weekly PSS-10 questionnaires using JISC Online Surveys.

Participants allocated to the intervention group additionally completed an individual resonance frequency (RF) assessment. RF refers to each participant’s personalised breathing rate (the rate at which their individual HRV amplitude was maximised) and should be distinguished from the broader term “resonance frequency breathing” (RFB), which denotes the intervention approach. To determine each participant’s RF, breathing was paced from 7.0 to 4.5 breaths per minute in 0.5-breath decrements using the Paced Breathing application while HRV was continuously recorded, following established protocols (Lehrer et al., 2003; Shaffer and Meehan, 2020). RF was identified as the breathing rate associated with the highest SDNN and RMSSD values. To minimise hyperventilation and vagal withdrawal, final selection was based on a convergence of physiological indices and participant-reported comfort (Porges, 1995; Thayer et al., 2012; Lehrer et al., 2013). This rate was subsequently programmed as the default pacing for all training sessions.

#### 2.4.2 Phase 2: Training (weeks 1–3)

Participants in the intervention group were instructed to complete daily 10-min RFB sessions (21 total across 3 weeks) at their individually determined RF using the Paced Breathing application. Sessions were completed remotely in a seated position, in a quiet and comfortable environment, guided by visual and auditory pacing cues. Resting HRV recordings were collected three times per week for both intervention and control groups using the Elite HRV application on Monday, Wednesday, and Friday of each training week. Participants were instructed to complete HRV recordings within 10 min of waking, at approximately the same time each day, and prior to caffeine consumption to obtain consistent resting measures (Shaffer and Ginsberg, 2017). Each recording lasted 5 min, in accordance with Task Force of the European Society of Cardiology and the North American Society of Pacing and Electrophysiology (1996) guidelines, and was submitted immediately following completion via Microsoft Teams. Weekly HRV values were calculated as the mean of all valid recordings collected each week. Baseline HRV was obtained from a single five-minute resting recording collected during the familiarisation session under standardised conditions.

Perceived stress was assessed weekly using the PSS-10, completed virtually at the end of each training week. To support adherence and engagement, a brief remote check-in lasting approximately 10 min was conducted during Week 2, informed by evidence that brief human support can enhance adherence in digital interventions (Mohr et al., 2011). Adherence was self-monitored and reported via digital logs at study completion. Participants in the control group did not receive any intervention but followed the same HRV and PSS-10 data collection schedule throughout the study period.

#### 2.4.3 Phase 3: Follow-up (week 4)

Following completion of the training phase, participants in the intervention group completed a semi-structured interview during Week 4. Interviews were conducted remotely via Microsoft Teams, audio-recorded, and transcribed verbatim for qualitative analysis. Interviews explored participants’ experiences of the intervention, including feasibility, perceived impact, engagement, and future application.

### 2.5 Quantitative analysis

Quantitative analyses were conducted using Jamovi (Version 2.6; The jamovi Project, 2024). Descriptive statistics were reported as M (SD). HRV recordings were aggregated into weekly values by averaging all valid recordings within each week. If a recording was not received or failed quality screening, it was treated as missing and excluded from the weekly average, with no imputation applied. Change scores were calculated for each participant from baseline (Week 0) to each subsequent time point. Mann–Whitney U tests were used to compare between-group change scores at each time point. Given the pilot and exploratory nature of the study, no formal correction for multiple comparisons was applied; analyses were intended to provide preliminary estimates of effect size and direction to inform the design of a subsequent adequately powered trial rather than to serve as confirmatory hypothesis tests. The implications of this approach are addressed in Section 4.7. Spearman rank-order analyses examined associations between changes in HRV indices (SDNN, RMSSD) and changes in perceived stress (PSS-10). Given the small sample size and the limited reliability of normality testing in pilot HRV data, non-parametric tests were used throughout. Effect sizes were calculated using the rank-biserial correlation (r) for Mann–Whitney U tests.

### 2.6 Interview guide

A semi-structured interview guide was used to explore participants’ experiences of the RFB intervention, focusing on feasibility, perceived impact, engagement, and future application. This format ensured consistency across interviews while allowing flexibility to examine individual experiences (Kallio et al., 2016). The guide was informed by the study aims and literature on stress regulation and HRV biofeedback (Ghasemi et al., 2024; Lehrer and Gevirtz, 2014). Questions addressed the practicality of the 10-min daily protocol, perceived psychological and physiological effects, factors influencing engagement and adherence, and intentions for continued use. Open-ended questions were supplemented with probing prompts (e.g., “*Can you tell me more about that?*”) to encourage elaboration and clarify responses. Interviews were conducted individually via Microsoft Teams, lasted approximately 20–30 min, and were audio-recorded in accordance with participant consent.

### 2.7 Qualitative analysis

Qualitative data were analysed using reflexive thematic analysis, guided by Braun and Clarke’s (2006, 2021) six-phase framework. A deductive orientation was adopted, with *a priori* analytic constructs developed in relation to the study’s evaluative aims: feasibility, perceived impact, engagement, and future application of the RFB intervention. These constructs were informed by relevant literature and theoretical considerations (e.g., stress regulation and behavioural adherence). Transcripts were coded manually to facilitate close engagement with the data, and themes were iteratively refined to ensure analytic clarity. Although analysis was structured around predefined areas of interest, reflexivity was maintained throughout to acknowledge the researcher’s interpretive role and to allow identification of unanticipated insights.

### 2.8 Integration strategy

In line with the convergent mixed-methods design, quantitative and qualitative data were analysed independently and interpreted in parallel to enable triangulation (Creswell and Creswell, 2018). Integration occurred during interpretation, allowing physiological outcomes and subjective experiences to be considered jointly. This strategy was intended to leverage the complementary strengths of both data strands, such that where quantitative outcomes were limited in sensitivity or statistical power, qualitative data could illuminate participant experiences and perceived benefits not fully captured by global self-report measures such as the PSS-10 (Fetters et al., 2013; Guetterman et al., 2015).

### 2.9 Ethical considerations

Ethical approval was granted by the Moray House School of Education and Sport Ethics Committee at the University of Edinburgh. Participants provided written informed consent prior to participation. Data were pseudonymised, securely stored, and accessible only to the research team. Interview recordings were deleted following transcription verification. All procedures complied with UK GDPR and the British Psychological Society (2021) Code of Human Research Ethics.

## 3 Results

Table 4 presents descriptive and inferential statistics, with change scores illustrated in Figure 1.

**Table 4:** HRV and PSS-10 change scores across the intervention period.

Variable	Week	Intervention, M (SD)	Control, M (SD)	*p*	*r*
SDNN (ms)	B-W1	7.58 (11.2)	−1.32 (31.2)	0.310	0.39
	B-W2	25.0 (24.9)	−14.3 (17.2)	0.004*	0.94
	B-W3	37.1 (23.9)	−15.1 (26.6)	0.026*	0.78
RMSSD (ms)	B-W1	24.7 (37.4)	−9.4 (50.7)	0.240	0.44
	B-W2	32.0 (40.3)	−19.6 (50.5)	0.132	0.56
	B-W3	60.4 (62.2)	−19.4 (47.3)	0.026*	0.78
PSS-10	B-W1	−1.50 (7.12)	1.67 (3.01)	0.809	–0.11
	B-W2	1.50 (10.10)	3.67 (7.39)	0.421	–0.31
	B-W3	−6.00 (12.20)	4.50 (7.42)	0.199	–0.47

Data are presented as M (SD) of change scores from baseline to each intervention week (B–W1, B–W2, B–W3). For PSS-10, negative change scores indicate reductions in perceived stress relative to baseline; for HRV indices, negative change scores indicate reductions in HRV. All between-group comparisons used Mann–Whitney U tests. r represents the rank-biserial correlation effect size, with positive values indicating greater increases in HRV, and negative values indicating greater reductions in perceived stress, in the intervention group relative to controls. *p < 0.05.

**Figure 1:**
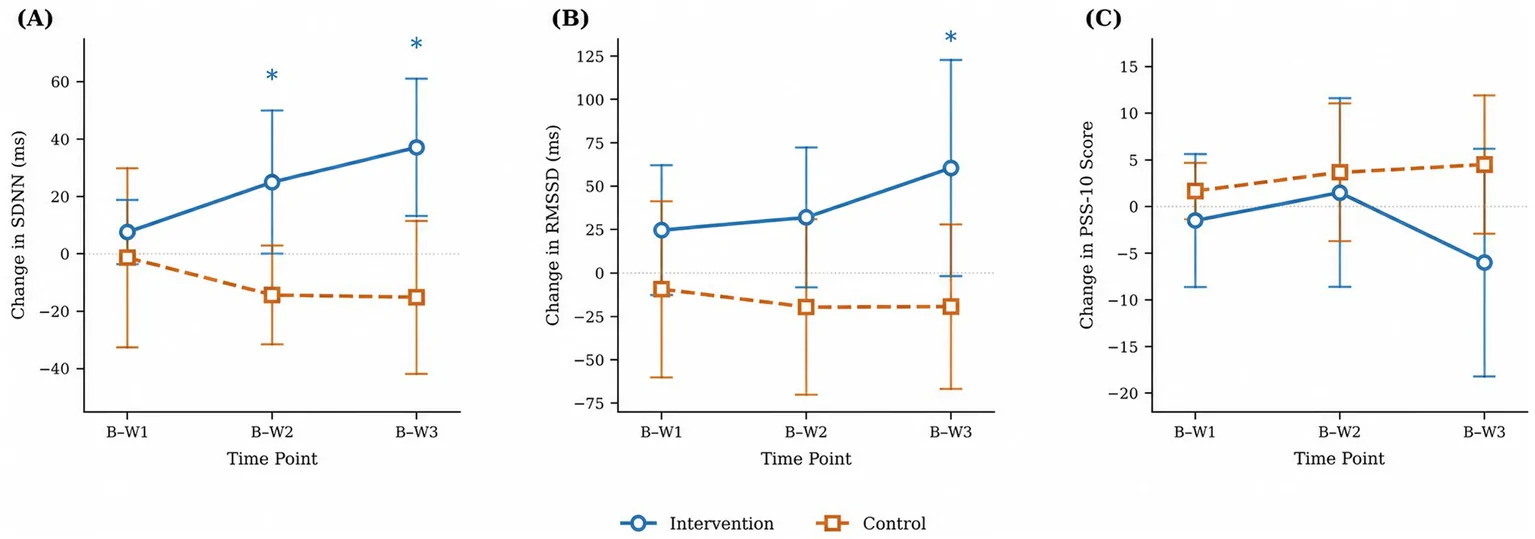
Mean change scores for HRV and perceived stress. Panels **(A–C)** present mean (SD) change scores from baseline to each intervention week (B–W1, B–W2, and B–W3) for the Intervention and control groups. Panel **A** = SDNN; Panel **B** = RMSSD; Panel **C** = PSS-10. Error bars represent ±1 SD; bars extending beyond the axis limits are truncated for display clarity. Positive change scores indicate increases from baseline, whereas negative change scores indicate decreases. *p < 0.05 for between-group comparisons.

### 3.1 Heart rate variability—H1

HRV parameters increased in the intervention group, whereas the control group demonstrated declines across the intervention period. Mann–Whitney *U* tests indicated significant between-group differences in change scores for SDNN at Week 2 (*U* = 1.00, *p* = 0.004, *r* = 0.94) and Week 3 (*U* = 4.00, *p* = 0.026, *r* = 0.78), and for RMSSD at Week 3 (*U* = 4.00, *p* = 0.026, *r* = 0.78). H1 was supported.

### 3.2 Perceived stress—H2

No statistically significant between-group differences were observed for perceived stress at any time point (all *p* > 0.05). However, change scores diverged across the intervention period. The intervention group showed a net reduction in perceived stress by Week 3. The Week 2 value represents an increase, so the trajectory was not progressively declining, whereas the control group showed a consistent increase. By Week 3, this divergence yielded a moderate effect size, with the intervention group demonstrating a mean decrease of 6.00 points from baseline (M = −6.00, SD = 12.20) and the control group a mean increase of 4.50 points (M = +4.50, SD = 7.42). H2 was not supported.

### 3.3 Correlation analysis—H3

Spearman rank-order correlations were calculated using Week 3 change scores within the intervention group to evaluate associations between HRV indices and perceived stress. No significant correlations were observed between RMSSD and PSS-10 (*ρ* = 0.03, *p* = 0.957) or between SDNN and PSS-10 (*ρ* = –0.14, *p* = 0.787). H3 was not supported.

### 3.4 Qualitative analysis—H4

Interviews were completed by five of the six participants in the intervention group. All interviewed participants (5/5) described perceived benefits in stress coping, four noted feasibility of the 10-min format, three described increased focus or mental clarity, and two mentioned intentions for continued use after the study. Each theme is discussed below and illustrated with direct participant quotations. H4 was supported.

#### 3.4.1 Feasibility

Most participants found the 10-min duration achievable: “I think it was the right amount because if it had been 15 min or 20 min, it would have not been manageable… So, 10 min I would say is a good amount.” (P06). However, several participants noted some initial challenges during the first week: “At first, I was like, oh, is this going to be a little bit too long? But once I started doing it, I found that 10 min was a good amount of time…” (P12). Another participant mentioned a desire for greater flexibility in session length due to scheduling issues: “Maybe I would have done 5 min if I’m super busy… but overall, I think 10 min was perfectly fine.” (P08).

#### 3.4.2 Perceived impact

Participants noted positive changes in stress regulation and psychological functioning. Many described reduced reactivity to stressors. One participant stated, “I wasn’t as quick to react… I felt a lot more in control of myself and my emotions.” (P12). The breathing sessions supported clarity and focus: “I think they may have helped me more on a subconscious level… I was starting off my day with breathing exercises and that kind of helped me clear my mind and have a fresh perspective every day…” (P06). Participants also noted reduced duration and intensity of stress responses, suggesting improved stress recovery: “Instead of it [stressor] lasting 2 h, it lasted 30 min… I did not react as much as I normally do.” (P08).

#### 3.4.3 Engagement

Engagement with the intervention was influenced by both intrinsic motivation and external support. One participant mentioned their interest in physiological outcomes contributed to initial motivation: “I wanted to see the results. I was interested in seeing how this would impact my stress levels and HRV readings.” (P06). Reminders and researcher check-ins were key adherence facilitators: “Definitely the reminders… Like, oh yeah, I have something nice to look forward to… having myself check in with you helped me stay on track.” (P08). However, time constraints challenged engagement: “It’s hard for me to just block out and allocate time… I’m always thinking about the next thing I have to do.” (P12).

#### 3.4.4 Future application

Participants expressed intentions for continued use of RFB and supported its broader application in academic contexts. The protocol was viewed as a practical strategy for daily stress management: “Even after the study, I still do some breathing exercises… Even if it’s during the day when I’m by myself and I face a stressor… I would continue using this in the future.” (P08). One mentioned the value of adapting practice times: “Oh yes, absolutely. I probably would do it in the evening. Just adapting it to my preferences and my schedule.” (P08). Participants viewed potential integration within university wellbeing services positively: “I would take it up… when there’s a lot of uncertainty, a lot of stress in a person’s life… like dissertation time.” (P09).

### 3.5 Summary of results

HRV increased in the intervention group relative to controls, with significant between-group differences in change scores. No significant between-group difference in perceived stress was observed. Changes in HRV were not significantly correlated with changes in perceived stress, and qualitative findings supported the feasibility and acceptability of the protocol.

## 4 Discussion

The present study evaluated the preliminary effects, feasibility, and acceptability of a brief, app-guided resonance frequency breathing (RFB) intervention among postgraduate students during heightened academic demand. HRV increased in the intervention group relative to controls, with significant between-group differences (H1). Qualitative findings and self-reported adherence supported the protocol’s feasibility and acceptability (H4). However, no significant between-group difference in perceived stress change was observed (H2), and changes in HRV were not significantly correlated with changes in perceived stress (H3).

### 4.1 HRV and autonomic regulation—H1

Participants in the RFB group showed increases in both SDNN and RMSSD relative to controls, reflecting enhanced resting HRV. SDNN indexes overall variability in cardiac intervals, capturing combined sympathetic and parasympathetic influences, whereas RMSSD reflects short-term, vagally mediated cardiac control (Shaffer and Ginsberg, 2017). In short-term recordings such as those used here, SDNN is substantially influenced by vagal activity. The concurrent increase across both indices suggests a broader HRV response to the intervention rather than an isolated change in a single parameter.

Between-group differences in change scores from baseline were significant for SDNN at Weeks 2 and 3, whereas the difference for RMSSD was significant only at Week 3. The differing time points may indicate that SDNN and RMSSD varied in their sensitivity to intervention-related change; however, this timing cannot establish a sequence of autonomic adaptation. More broadly, the observed increases are consistent with the baroreflex resonance model, which proposes that breathing at approximately 0.1 Hz amplifies respiratory sinus arrhythmia and stimulates the baroreflex, while repeated practice can increase resting baroreflex gain (Lehrer et al., 2003; Vaschillo et al., 2006).

In contrast, HRV declined in the control group across the same period, a pattern also observed during examinations in student populations (Hammoud et al., 2019). This decline is consistent with findings from Benítez-Agudelo et al. (2025), who observed non-significant downward trends in several HRV indices across an academic semester alongside significant increases in anxiety and depressive symptoms. In their data, higher baseline HRV predicted better academic performance, highlighting the potential functional value of preserving autonomic flexibility. The maintenance and increase of HRV in the intervention group may reflect a buffering effect, whereby regular RFB practice attenuated stress-related reductions in autonomic function.

### 4.2 Perceived stress outcomes—H2

Despite HRV improvements, perceived stress change scores did not differ significantly between groups. Several factors may account for this null finding. First, the small sample size (*N* = 12) limited the ability to detect modest psychological effects. Although non-significant, PSS-10 scores trended in the hypothesised direction. By Week 3, intervention participants showed a mean decrease of 6.00 points from baseline while controls showed a mean increase of 4.50 points. This corresponded to a moderate effect size (*r* = −0.47, *p* = 0.199), suggesting that the observed difference merits further investigation. The observed 6-point reduction may represent a potentially meaningful change, though this requires confirmation in larger samples.

Second, the PSS-10 is a global, retrospective measure and may lack sensitivity to short-term or context-specific psychological changes. Furthermore, the adapted one-week recall period used in the present study departs from the original one-month format for which PSS-10 psychometric properties were established (Cohen and Williamson, 1988); this modification may have reduced sensitivity or reliability. This should therefore be considered an additional explanation for the absence of significant psychological effects.

A third explanation concerns intervention duration. Although the present protocol employed shorter daily sessions than those used by Chaitanya et al. (2022), session duration is unlikely to explain the null finding. Brief breathing practices can yield psychological benefits (Balban et al., 2023), and session duration was not associated with effectiveness among practices lasting at least 5 min (Bentley et al., 2023). Overall intervention duration may nevertheless have been a constraint. Blase et al. (2021) reported that psychological benefits were visible after four weeks of HRV biofeedback training and described eight weeks of daily practice as more promising, although evidence regarding optimal duration remains mixed.

### 4.3 HRV and perceived stress—H3

Changes in HRV were not significantly correlated with changes in perceived stress. Given the limited statistical precision, the nonsignificant association should not be interpreted as evidence of a dissociation between physiological and psychological outcomes. Rather, the present study did not provide evidence that changes in these outcomes covaried over the three-week intervention. Across the studies in their meta-analysis, Donnelly et al. (2023) reported improvements in both HRV and depressive symptoms, but the magnitudes of these effects were not significantly associated. One possible explanation is that physiological and psychological responses unfold over different timescales. RFB is designed to act directly on cardiorespiratory and baroreflex regulation, whereas vagal afferent pathways provide a route through which peripheral autonomic activity may influence central networks involved in cognition and affect (Porges, 2007). Changes in stress appraisal or perceived stress may require longer or more sustained practice. Consistent with this possibility, Galin and Keren (2024) describe possible pathways through which autonomic changes may precede and subsequently influence emotional processes. Huang et al. (2018) found stronger longitudinal evidence that lower HRV predicted later depressive symptoms than for the reverse pathway. However, these findings concern depression over substantially longer periods and do not establish that autonomic change preceded psychological adaptation in the present study. These data cannot determine whether psychological changes follow physiological change or unfold independently, nor can they exclude the possibility that an association between the outcomes was obscured by limited statistical precision. Longer trials with follow-up assessments are needed to examine the temporal relationship between these responses.

### 4.4 Feasibility, engagement, and user experience—H4

Qualitative findings supported the feasibility of the RFB protocol. Participants reported perceived psychological benefits, including enhanced calmness, reduced emotional reactivity, and increased self-awareness. These accounts may help contextualise the contrast between the HRV and PSS-10 findings by identifying perceived changes that may not have been captured by a global retrospective measure. However, participants’ accounts do not independently establish an intervention effect. Rather, they provide complementary evidence concerning how the intervention was experienced, consistent with a mixed-methods integration framework (Fetters et al., 2013). Future studies may incorporate ecological momentary assessment (EMA) to reduce recall bias and better capture within-day fluctuations and immediate responses to the intervention (Shiffman et al., 2008).

Participants described the 10-min daily sessions as manageable and compatible with demanding dissertation schedules. Self-reported adherence was 81%, indicating that most prescribed sessions were completed. This provides useful feasibility information because adherence is often poorly documented in home-based breathing interventions (Vlemincx and Cortez-Vázquez, 2024). Brief reminders and a scheduled mid-intervention check-in functioned as accountability mechanisms. This is consistent with Mohr et al.’s (2011) supportive accountability model, which proposes that human support can promote adherence to digital interventions through accountability. Participants reported intentions to continue RFB after the study, with one individual noting continued use during stressful moments. These accounts support the protocol’s acceptability, but no follow-up data were collected to determine whether continued practice was sustained.

### 4.5 Implications

Whereas many RFB interventions require substantial daily practice and extended training periods, the present study detected changes in resting HRV following 10 min of daily RFB over 3 weeks using a freely available smartphone application. By targeting cardiorespiratory and baroreflex regulation, RFB may offer a complementary approach to cognitively focused stress-management strategies. This may be particularly relevant for individuals experiencing chronic stress, given its association with lower resting-state HRV (Jiryis et al., 2022). In this respect, RFB protocols may broaden the range of stress-regulation strategies available within university settings and support autonomic regulation during intensive academic phases.

### 4.6 Methodological considerations

HRV was assessed using the Polar H10 chest strap, which has demonstrated strong agreement with electrocardiography for RR-interval recording at rest (Schaffarczyk et al., 2022). Recordings followed Task Force recommendations for short-term HRV assessment by using five-minute resting-state windows (Task Force of the European Society of Cardiology and the North American Society of Pacing and Electrophysiology, 1996). This standardised duration was appropriate for the study’s feasibility-focused design. The use of individually determined resonance frequency (RF) was consistent with established RFB protocols and may have contributed to the observed HRV changes (Lehrer et al., 2003, 2013).

### 4.7 Limitations and future directions

Several limitations warrant consideration. The small sample size limits statistical power, reduces precision, and limits generalisability. Replication in larger samples with adequate power is therefore necessary to detect more subtle psychological effects. Although large effect sizes were observed for HRV improvements, these findings should be interpreted as preliminary signals rather than definitive evidence of efficacy. Effect sizes should be interpreted with caution, as small sample sizes can produce inflated and unstable estimates that may not generalise to population-level effects, particularly for HRV outcomes characterised by high within-person variance. Between-group differences were assessed using Mann–Whitney U tests with rank-biserial effect sizes, and HRV–stress associations using Spearman correlations, given the small sample and limited ability to assess distributional assumptions.

Multiple statistical comparisons were conducted without formal correction, consistent with the exploratory aims of the pilot. However, this may have inflated the risk of false-positive findings. Repeated-measures models were not employed due to the small sample size and instability of variance estimates inherent in a pilot dataset, which would render such models underpowered and their assumptions difficult to satisfy. Future research should apply more robust analytical approaches, such as repeated-measures ANOVA or mixed-effects models to examine Group × Time interactions.

The control group did not receive an active intervention; therefore, non-specific effects of structured relaxation, expectancy, or engagement cannot be separated from resonance-frequency-specific effects. Future studies should include an active control condition, such as paced breathing at a non-resonant frequency, to better isolate the specific effects of RFB.

Participants were self-selected postgraduate students, introducing potential motivational bias, and the absence of follow-up assessments precludes conclusions regarding maintenance effects. Adherence was assessed via self-report and should be supplemented with automated usage tracking in future research (Balban et al., 2023).

Menstrual cycle phase was not recorded or controlled for in female participants, and given that eight of 12 participants were female, hormonal variability may have contributed to HRV differences across the intervention period (Schmalenberger et al., 2019; Yazar and Yazıcı, 2016). Future studies should consider recording cycle phase or stratifying analyses by hormonal status. This uncontrolled source of physiological variability may have contributed to within-group dispersion in RMSSD and SDNN.

Finally, HRV assessment was limited to the time-domain indices SDNN and RMSSD. Inclusion of frequency-domain and nonlinear HRV indices would provide a more comprehensive characterisation of autonomic responses to RFB. Moreover, while HRV is a well-established marker of autonomic function, it remains an indirect index; inclusion of complementary physiological measures, such as baroreflex sensitivity, would strengthen mechanistic inference.

## 5 Conclusion

This pilot study supports the feasibility and acceptability of a brief, app-guided RFB protocol involving 10 minutes of daily practice over 3 weeks in postgraduate students. Preliminary findings indicated that the intervention was associated with increased resting HRV. Perceived stress did not differ significantly between groups, although the estimated effect was moderate and in the hypothesised direction. Changes in HRV were not associated with changes in perceived stress. Qualitative findings suggested perceived improvements in stress coping. These findings support further evaluation in a larger, adequately powered trial with longer-term follow-up.

## Data Availability

The original contributions presented in the study are included in the article/supplementary material, further inquiries can be directed to the corresponding author.
